# DRNet: A Depth-Based Regression Network for 6D Object Pose Estimation

**DOI:** 10.3390/s21051692

**Published:** 2021-03-01

**Authors:** Lei Jin, Xiaojuan Wang, Mingshu He, Jingyue Wang

**Affiliations:** 1 School of Computer Science, Beijing University of Posts and Telecommunications, Beijing 100876, China; jinlei@bupt.edu.cn; 2 School of Electronic Engineering, Beijing University of Posts and Telecommunications, Beijing 100876, China; hemingshu@bupt.edu.cn (M.H.); jingyue0548@bupt.edu.cn (J.W.)

**Keywords:** 6Dof pose estimation, rotations, translations

## Abstract

This paper focuses on 6Dof object pose estimation from a single RGB image. We tackle this challenging problem with a two-stage optimization framework. More specifically, we first introduce a translation estimation module to provide an initial translation based on an estimated depth map. Then, a pose regression module combines the ROI (Region of Interest) and the original image to predict the rotation and refine the translation. Compared with previous end-to-end methods that directly predict rotations and translations, our method can utilize depth information as weak guidance and significantly reduce the searching space for the subsequent module. Furthermore, we design a new loss function function for symmetric objects, an approach that has handled such exceptionally difficult cases in prior works. Experiments show that our model achieves state-of-the-art object pose estimation for the YCB- video dataset (Yale-CMU-Berkeley).

## 1. Introduction

Detecting objects and estimating their poses [1] are critical steps for many 3D applications, such as autonomous driving [2,3,4], augmented reality [5,6,7], and robotic grasping [8,9]. Object poses consist of rotations and translations. The challenges of estimating object poses lie in changing lighting conditions, heavy occlusion, sensor noise, etc. Recently, methods based on RGB-D images have made great progress [4,10,11]. However, for many scenes, depth sensors are not available. Pose estimation based on RGB only remains a challenging problem. In this paper, we focus on 6D pose estimation using only a single RGB image.

Traditional methods, such as keypoint-based methods [12,13,14,15,16,17] and template-based methods [18], suffer from certain disadvantages. Depending on the rich texture information needed to recognize key points or extract features, these methods are unable to handle texture-less objects and occlusions.

**Figure 1:**
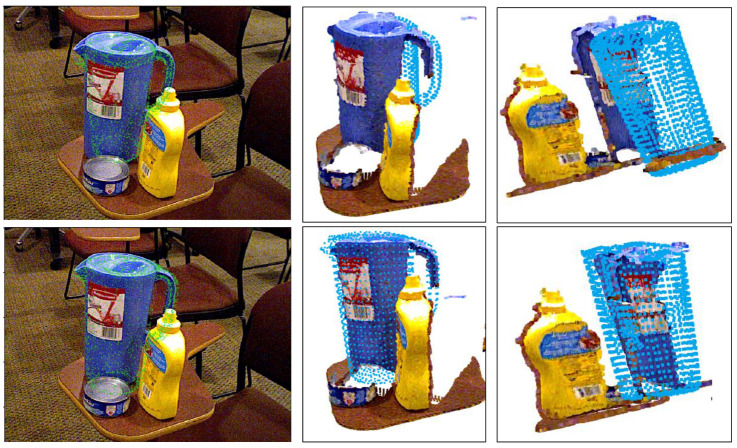
The Translation Error in Holistic Models. The first row shows translation errors in PoseCNN [20]. The second row shows examples of our model. It is difficult to show translation error from the 2D projection images. Therefore, we present the object in 3D point cloud from different perspectives. The blue point clouds are the predicted results. We found that our model performs better in translation.

Nowadays, methods based on the conventional neural network (CNN) have been developing rapidly. Compared with traditional methods, they are more suitable for common clustering scenes in daily life. Methods based on CNN can be divided into two classes. The first class is the two-stage method, which uses CNN to regress keypoints and then computes poses using the Perspective-n-Point (PnP) algorithm [19]. However, methods based on keypoints can not solve symmetric object pose estimations and occluded scenes. The second class is a holistic method for regressing poses directly. Nevertheless, a big challenge for end-to-end RGB methods is that it is hard to work out the translations of objects. Previous research [20,21] has regressed translations directly. Figure 1 indicates the translation errors in holistic models (the quantitative experimental results are presented in Section 4.3). The predicted point cloud shifts backward compared with the ground truth. It is the large searching space of translations that makes the network hard to converge. The prior works discussed are not considered to encode the depth information appropriately. If we have the depth of objects to produce the initial translation, it is possible to obtain more accurate results by reducing the searching space for the subsequent module. To handle this problem, we consider that techniques should be adopted from the depth estimation. We tackle the depth estimation through a coarse-to-fine process, which uses a classification network to produce coarse depth and refines the depth with a regression network. In addition, we find that holistic methods [10,20] where Average Distance of Model Points for Symmetric Object (ADD-S) loss is used to solve symmetric objects may cause particularly difficult cases. ADD-S loss is used to train symmetric objects, which is proposed by [20]. It is found that ADD-S loss is not suitable for every symmetric object.

In this work, we propose a generic method based on RGB inputs to solve the 6D pose estimation problem. Then, we focus on the particularly difficult cases caused by ADD-S loss. A new loss function is set to resolve the symmetric object pose estimation problem. From our visualizations, we find that particularly difficult cases are solved. Compared with other RGB methods, we achieved state-of-the-art object pose estimation for the YCB-video dataset (Yale-CMU-Berkeley). The innovations of this paper are shown as follows:

(1) To calculate translations of objects, we apply techniques from the depth estimation task to the pose estimation task. In addition, a depth-refined module is designed to obtain the accurate depth of objects for indoor scenes.

(2) A pose regression module is schemed to regress rotations and refine the initial translations produced by the translation estimation module.

(3) We design a method of synthesizing depth maps, by virtue of which our model training could be processed without the need to use ground truth depth maps.

(4) A new loss function, which is more suitable for symmetric objects, is put forward to replace ADD-S loss.

The rest of the paper is structured as follows, Section 2 introduces related works of our paper. Section 3 presents our method in details. The experimental results are shown in Section 4. Section 5 concludes our work.

## 2. Related Work

Poses from RGB inputs. Some previous methods obtain object poses by matching keypoints with known object models [22]. In contrast, some methods tend to use template-based techniques, which are easily affected by occlusion and varied environments [23,24]. In addition, two-stage methods detect keypoints and solve the pose estimation by PnP [19]. Suwajanakorn et al. [25] extracted 3D keypoints for recovering object poses. Mousavian et al. [26] obtained 3D bounding boxes on the challenging KITTI dataset by using geometric constraints of 2D-object bounding boxes. Pavlakos et al. [27] used semantic keypoints to make the 6-DoF object pose reappear for both instance-based and class-based scenarios with a cluttered background. To overcome truncation scenes, Peng et al. [28] proposed a Pixel-wise Voting Network (PVNet) to identify keypoints with the aid of RANSAC-based voting. However, methods based on keypoints are not capable of handling symmetric objects well, and suffer from occlusions. Recently, some CNN methods have aimed to obtain poses of objects in a single shot. Kendall et al. [29] constructed a Posenet to regress the 6D pose in an end-to-end manner, which is robust to difficult lighting and motion blur. Li et al. [30] proposed a pose refined framework based on RGB images, which iteratively refines the pose by matching the rendered image against the observed image. Billing et al. [21] put forward a SilhoNet which predicts an intermediate silhouette representation for objects to refine the results of pose regression. We find that, for RGB methods, it is harder to figure out translations than rotations. We advise to use the estimated depth of objects to produce initial translations, which can reduce the searching space for subsequent networks. Finally, it will be easier to predict the offsets of initial translations than predicting translations directly.

Depth estimation is a basic task for understanding 3D scenes. Early works [31,32,33] used geometry-based algorithms that relay on point correspondences between images and triangulation to estimate distance. With the development of CNN, supervised depth estimation methods have been proposed. Guler et al. [34] integrated ideas from semantic segmentation with regression task, which improves the performance of the depth estimation. Laina et al. [35] used residual learning and multi-layer deconvolutional networks to recover depth. Roy et al. [36] combined random forests and convolution neural networks to obtain more accurate depth maps. Methods such as stage-wise refinement [37,38] and skip-connection strategies [39] have also been adopted in depth estimation tasks. Fu [40] discretized depth and obtained final results by virtue of ordinal regression, leading to the most advanced supervised depth estimation work carried out thus far. Semi-supervised and unsupervised methods [41,42] have also been proposed. It can be observed that most methods focusing on relative distances are not suitable for our task. In contrast, our method uses Deep Ordinal Regression Network (DORN) [40] as a part of the translation estimation module. It is important that we only concentrate on the area on known objects. That is why some modifications are necessary when this method is applied to pose estimation tasks.

## 3. Method

Our goal is to estimate the 6D pose of several known objects with an RGB image of a cluttered scene. As in other other methods, we represent the 6D pose as a rotation R∈SO(3) and a translation $t\in {ℜ}^{3}$. The object 3D models are available and the object coordinate system is defined in the 3D space of the model. Moreover, 6D poses are defined with respect to the camera coordinate frame.

In this paper, we argue that it is harder to estimate *t* than *R* from an RGB image. How to produce an appropriate initial *t* is a key problem to figure out. Furthermore, estimating 6D poses from an RGB image is a process that suffers from heavy occlusions, poor lighting, and other obstacles.

We tend to address the above problems with a translation estimation module and a 6D poses regression module. For translation estimation, we adopt the state-of-the-art Deep Ordinal Regression Network (DORN) in supervised depth estimations. In addition, we add a depth refined module behind the DORN for more accurate depth (Section 3.3). Subsequently, poses are obtained through a pose regression module (Section 3.4). Finally, we point out in Section 3.4 that ADD-S loss can result in particularly difficult cases for symmetrical objects. Therefore, a new loss function is adopted in our paper. A method of synthesizing depth maps is introduced in Section 3.5.

### 3.1. Architecture Overview

**Figure 2:**
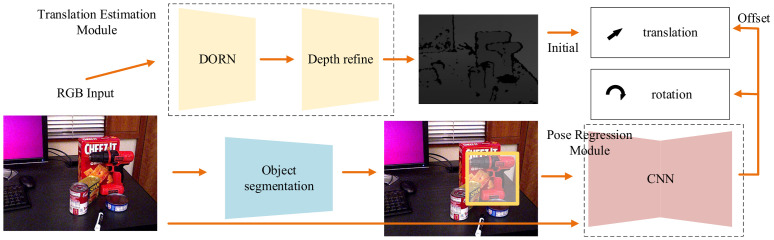
Overview of Our 6D Pose Regression Network. Our network generates object bounding boxes and masks through a segmentation network. The translation estimation module produces initial *t*, and CNN is a pose regression module, which produces *R* and the offset of *t*.

The overview of our 6D pose regression network is shown in Figure 2. First, an object segmentation network gives the bounding boxes and masks for objects in images. The translation estimation module takes color images as inputs and predicts initial translations for objects. Then, for each segmented object, we feed an image patch cropped by the bounding box of the mask and the original image into the pose regression module. The pose regression module extracts features from the Region of Interest (ROI) and original images, respectively. Afterwards, it concatenates these features and predicts the translation offset and the rotation for each object. The initial value of the translation is provided from the depth map predicted by the translation estimation module. The details of our architecture are described below.

### 3.2. Object Segmentation

As in other methods, object segmentation is first executed to obtain the ROI of objects. The segmentation module outputs N+1 channelled semantic segmentation maps with an RGB image. Each channel of *N* channels is a binary mask for each known object. One more channel presents the background. Our paper mainly discusses the 6D pose regression. Thus, the segmentation architecture proposed by [20] is employed.

### 3.3. Translation Estimation Module

For 6D poses, our translation estimation module adopts techniques from traditional Depth Estimation tasks [35,36,37]. However, our work is a little different from the depth estimation. On one hand, we only pay attention to the area of specific objects in the pictures. It is easier simply to measure the depth of specific objects in the images. On the other hand, for indoor scenes, we need more accurate depth to obtain better initial translations. A depth refined module is accordingly designed to obtain more accurate depth. In general, we apply the techniques of depth estimation to predict an initial translation in the translation estimation module.

**Figure 3:**
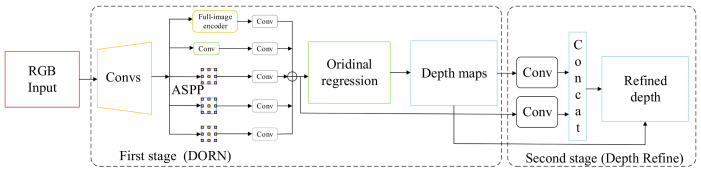
The Architecture of Translation Estimation Module. Convs represents ResNet50. Conv is the combination of conv2d, BN, Relu. The first stage is DORN. The second stage is Depth refine.

Figure 3 illustrates the details of translation estimation module. The first stage is the same as DORN [40]. We merely reduce the channel number to prevent time consumption and overfitting. The second stage is our depth refined module. We concatenate embedding features from the first stage and the predicted depth map to obtain the offset between the ground truth depth map and the predicted depth map. Next, we add the offset map obtained by the second stage to the depth map predicted by the first stage.

DORN discretizes depth and recasts depth network learning as an ordinal regression problem. Here, we discretize depth as the uniform distribution.

(1) $$h_{i}=\alpha +\left(\beta -\alpha \right)*d_{i}^{gt}/K,$$

The node *i* is an image pixel. $h_{i}$ is the discretization threshold for the node *i*. $d_{i}^{gt}$ is the depth of the node *i*. α is the minimum depth value. β is the maximum depth value. *K* is a constant. We set K=80 as [40] in this paper.

At the first stage, we use the ordinal loss used in [40] to update the network weight. The difference is that we only calculate the loss on the depth of objects in images. Next, a coarse depth map $d_{c}$ is generated and sent to the second stage with the embedding features from the ASPP [43].

The goal of the second stage is to correct the error caused by discretized depth values during the first stage. The challenge here rests with training the network to refine the previous prediction as opposed to making new predictions. Correspondingly, we should send the predicted depth map to the network. The predicted depth map is processed by 2D-convolutions to extract features. The embedding features from the first stage are processed by 1×1 2D-convolutions to reduce channels. Then, the above features are concatenated to predict the depth offset map. The loss used in the second stage is

(2) $$l_{refine}=\frac{1}{sum(M)}\sum ({d^{gt}}_{i}-d_{i})*M{}_{i},$$

*i* represents a pixel in maps. The ground truth depth map is $d^{gt}$. *M* is the mask of the image, in which 1 represents the foreground and 0 represents the background. *d* is the predicted depth. In addition, $d=d_{c}+\delta$. $d_{c}$ is the coarse depth map generated by the first stage. δ is the depth offset map predicted by the second stage.

We train different stages with different loss functions. The first stage is trained by the ordinal loss. The second stage is trained by $l_{refine}$. After getting refined depth maps *d*, we can obtain the initial translation of objects with the method as below. If the depth of a pixel in an image is obtained, then, according to the principle of a camera, we are able to obtain the coordinates of the pixel in space.

(3) $$\left[\begin{array}{c} x{}_{i}^{\prime }\\ y{}_{i}^{\prime } \end{array}\right]=\left[\begin{array}{c} f_{x}\frac{x_{i}}{d_{i}}+c_{y}\\ f_{y}\frac{y_{i}}{d_{i}}+c_{x} \end{array}\right],$$

Here, $x_{i},y_{i},d_{i}$ are the space coordinates with respect to the camera coordinate frame. $d_{i}$ is depth we obtain from the translation estimation module. $c_{y},c_{x}$ are the image size parameters. $f_{x}$,$f_{y}$ are the camera focal length. $x{}_{i}^{\prime },y{}_{i}^{\prime }$ are the coordinates of the pixels in the picture. According to the Formula (3), $x_{i},y_{i}$ can be calculated. Finally, we sample *n* nodes for every object in the image and obtain the initial translation for objects by using Formula (4).

(4) $$\left[\begin{array}{c} x\\ y\\ z \end{array}\right]=\frac{1}{n}\sum_{i=1}^{n}\left[\begin{array}{c} x_{i}\\ y_{i}\\ d_{i} \end{array}\right],$$

x,y,z are the initial translation for an object in the image. In this paper, we set *n* as 500.

### 3.4. Pose Regression Module

**Figure 4:**
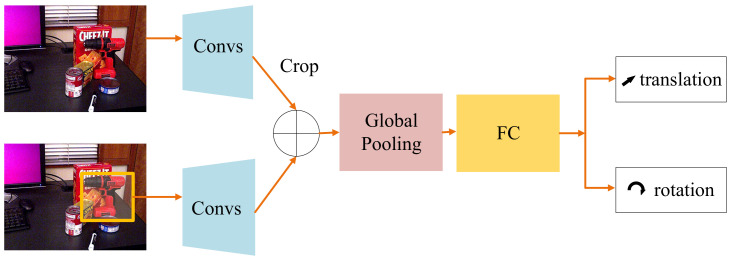
The Architecture of Pose Regression Module. The inputs of this module are the original image and the Region of Interest (ROI) of the image. Convs is ResNet18. The plus sign is concatenating operation. We use a global pooling to integrate information. A Fully Connected layer (FC) is used to regress 6D poses.

From Section 3.3, we obtain initial translations of objects in images. A pose regression module provides accurate rotations and translations based on initial translations. Figure 4 shows the architecture of our pose regression module. Obviously, only using the ROI makes it hard for the network to figure out translation offsets. To enrich context information, the original image is transmitted to another backbone (ResNet18 [44]). We crop the corresponding area from the feature map produced by the original image. By concatenating two feature maps together, the information from the context is fused with that from the objects. Global average pooling is used to integrate information. Finally, a Fully Connected (FC) layer regresses *R* and *t* offset, respectively.

After defining the module structure, we now concentrate on the learning target. We refer to previous research [10,20] which used the Average Distance of Model Points (ADD) loss. The ADD loss is defined as the distance between the points sampled on the objects model in ground truth pose and corresponding points on the same model transformed by the predicted pose. For asymmetric objects, the ADD loss is specified as

(5) $$l_{add}=\frac{1}{S}\sum_{i}∥\left(R\theta_{i}+t\right)-\left(\hat{R}\theta_{i}+\hat{t}\right)∥,$$

$\theta_{i}$ is the *i*th point of the S randomly selected 3D points from the object’s 3D model. $p=\left[R|t\right]$ is the ground truth pose. $\hat{p}=\left[\hat{R}|\hat{t}\right]$ is the predicted pose. For symmetric objects, ADD-S loss is

(6) $$l_{adds}=\frac{1}{S}\sum_{i}\min_{0<k<S}∥\left(R\theta_{i}+t\right)-\left(\hat{R}\theta_{k}+\hat{t}\right)∥.$$

**Figure 5:**
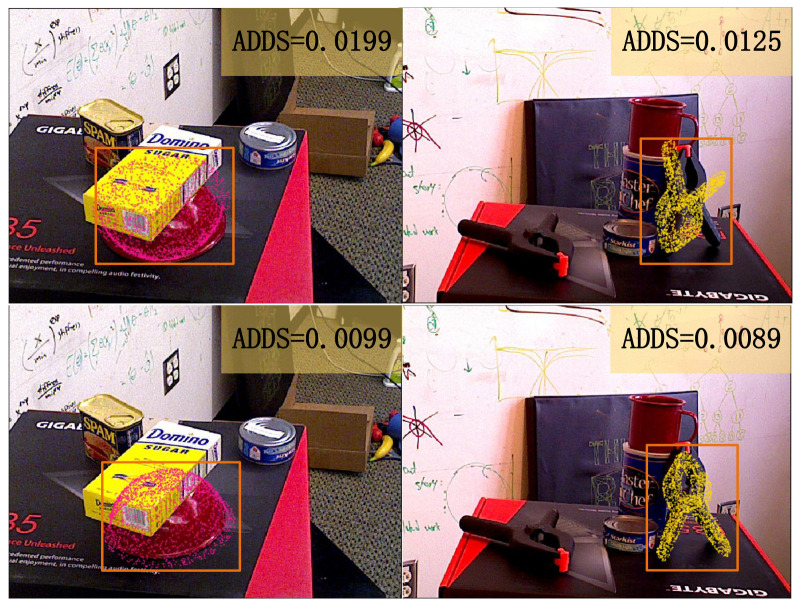
The particularly difficult Cases of Symmetric Objects. The first row are bad cases trained by ADD-S loss. The second row are examples of our model. The bowl and the clamp in the first row are found to be flipped. However, the ADD-S is small (<2 cm) for these bad cases. Here, the measuring unit we use is m. In addition, the 2 cm metric is utilized in [10].

However, if we use the Average Closest Point Distance (ADD-S) loss for symmetric objects, we will find particularly difficult cases in Figure 5. It is shown that the bowl and the clamp in the first row are flipped and the Average Closest Point Distances (ADD-S) are small. Two videos have bowls in the YCB test dataset [20]. The bowls in one of the videos are all flipped. Particularly difficult cases do not appear in just a few frames. The reason for this is that ADD-S loss simply calculates the nearest distances between points in two-point clouds. The model may achieve a local optimum. Then, ADD-S is not considered to be suitable for every symmetric object. To handle these bad cases, we propose a new loss function. For symmetric objects, symmetric axes are a must. Objects are consistent in 2D images when rotating about symmetric axes for a certain angle. This is a characteristic of symmetry and called as symmetric rotations. Consequently, we design a new loss function based on symmetrical axes of objects and take the minimum average distance between the points sampled on the objects model transformed by the ground truth pose plus the symmetric rotation and corresponding points on the same model transformed by the predicted pose. The new loss is defined as

(7) $$l_{addr}=\frac{1}{S}\min_{R^{\prime }}\sum ∥\left(RR^{\prime }\theta_{i}+t\right)-\left(\hat{R}\theta_{i}+\hat{t}\right)∥.$$

Here, $p=\left[R|t\right]$ is the ground truth pose. $p=\left[\hat{R}|\hat{t}\right]$ is the predicted pose. $R^{\prime }$ is the symmetric rotations. Different objects have different symmetric axes and symmetric rotations. Taking the bowl as an example, it has one symmetric axis and remains consistent after rotating arbitrary angle about the symmetric axis. For the clamp or the wood block, they can only rotate 180 degrees about the symmetric axis to remain consistent.

### 3.5. Synthetic Depth Map

When we collect 6D pose data, we may not have depth sensors. Besides, ground truth depth maps of datasets are sometimes inaccurate, and may suffer from the lack of depth caused by the character of infrared depth cameras. Our goal is to make our method training without using ground truth depth maps which are collected by depth cameras. Hence, we propose a depth data synthesis process.

Firstly, we have 3D object models. Besides, ground truth poses are already known. With ground truth poses, points of object models can be transformed to the coordinates in the camera frame. Subsequently, we can project points of 3D object models to images by using the Formula (3) with ground truth poses. The depth of points in 2D-images correspond to *z* coordinates of points which are transformed by ground truth poses. One important note: for most scenes, 3D object models are two-sided. The minimum *z* coordinates should be used for depth maps.

We train our translation estimation module and pose regression module with synthetic depth maps. Therefore, our model finishes the 6D pose training process without the aid of ground truth depth maps. It is an effective method for many scenes where depth maps are hard to obtain.

### 3.6. Training and Architecture Details

For the translation estimation module, the output channel number of ASPP is 1024. Then the following Conv is the combination of conv2d, BN and Relu. For the pose regression module, the output channel number of Convs is 256.

We first train our translation estimation module using the ground truth depth maps or the synthetic depth maps. Then we use the depth maps predicted by our translation estimation module to give an initial offset using the Formula (4) in our paper. The pose regression module is trained based on the initial offset predicted by our translation estimation module. The two modules are trained with the input-output relationship. The Adam optimizer is adopted. The learning rate is set as 1 × 10${}^{-4}$ for two modules.

## 4. Experiments

In this section, we attempt to verify the effectiveness of our method. The ablation studies are carried out on the ground truth maps (Section 4.3). In addition, the performance of synthetic depth maps is presented in Section 4.4. Section 4.5 compares our method with others The validity of our new loss is discussed in Section 4.6.

### 4.1. Datasets

YCB-Video Dataset. The YCB-Video Dataset is collected by Xiang [20]. The Dataset contains 92 RGB-D videos which are composed with 21 objects of varying shape and texture. The videos are annotated with poses and segmentation masks. Following previous methods [10,20], we divide the dataset containing 80 videos for training, 2949 key frames chosen from the rest of 12 videos for testing, and 80,000 synthetic images released by [20] in the training set.

Linemod Dataset. The Linemod Dataset consists of 13 low-textured objects in videos. The dataset is challenging due to varying lighting and clustered scenes. We render 10,000 images for each object in the Linemod dataset as [28].We further synthesize another 10,000 images using the “Cut and Paste” strategy. The background of each synthetic image is randomly sampled from SUN397 [45]. We also apply online data augmentation including random cropping, resizing, rotation, and color jittering during training.

T-LESS dataset. For T-LESS, we use 30 K physicallybased rendered (PBR) images from SyntheT-LESS, 50 K images of objects rendered with OpenGL on random photographs from NYU Depth V2 [46] and 38 K real images from [47]. In addition, we replace the background with random photographs.

### 4.2. Evaluation Metrics

For the comparison with other methods, we adopt the Average Distance of Model Points (ADD) metric for evaluation, following [10,20]. The definition of ADD is same as in Formula (5). It calculates the mean distances between model points transformed by ground truth poses and predicted poses. With regard to symmetric objects, we use the Average Closest Point Distance (ADD-S) in Formula (6) and report the area under the ADD(S) curve (AUC), doing the same as PoseCNN [20]. We set the maximum threshold of AUC to be 0.1 m as in previous papers. This measurement is universal for the YCB-Video Dataset.

In terms of the Linemod Dataset, we adopt the Average Distance of Model Points (ADD) for the asymmetric objects and the Average Closest Point Distance (ADD-S) for the symmetric objects, imitating prior works [14,48,49].

### 4.3. Ablation Study

In this section, we conduct ablation studies to verify the effectiveness of each module in our method. We execute our ablation study on the YCB-video dataset. Table 1 summarizes the results of ablation studies. Here, we calculate the ADD(S) AUC for the objects in the YCB-video dataset. For non-symmetric objects, we use the ADD and calculate the ADDS for symmetric objects.

**Figure 6:**
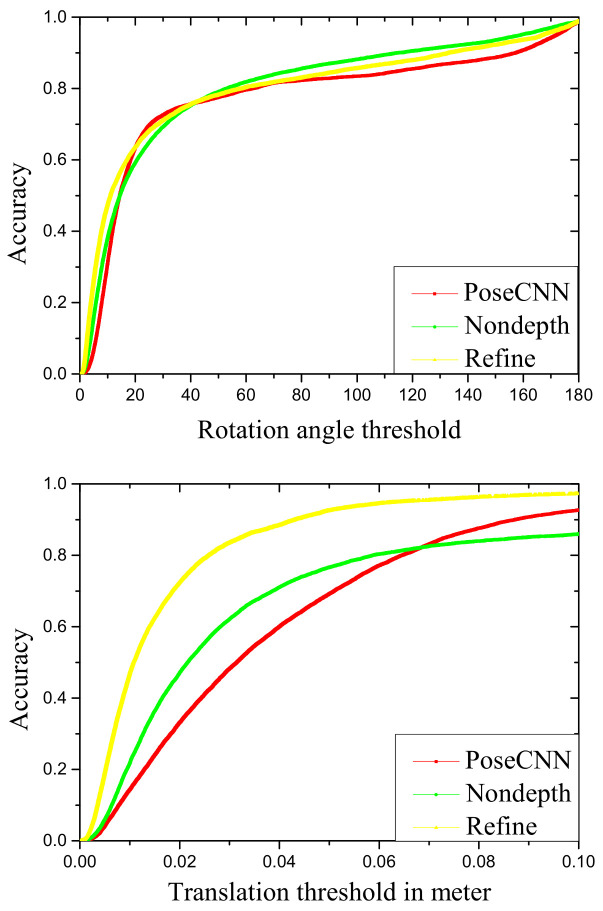
Accuracy of Rotations and Translations.

Effect of the translation estimation module. To analyze the translation estimation module discussed in Section 3.3, we conduct 6D poses experiments by using only the pose regression module. Hence, the initial translation *t* is zero. Compared with the result out of refined depth, the result is worse because the searching space is large. If we have the depth of objects to generate initial translations, it is easier for the network to regress offsets than predict the translations directly. Figure 6 shows the accuracy curves of rotations and translations. The performance of rotations in PoseCNN [20], Nondepth, and Refine is similar. Symmetric objects are meaningless to calculate the rotation accuracy. This cannot present the performance of our symmetric loss. Furthermore, the translation estimation module can improve the performance of translations to a large extent. It is confirmed that initial translations can reduce the searching space for the network, which is able to improve the performance of the network. The main difficulty of RGB methods lies in translations. Our method enhances the performance of translations.

Effect of the depth refine module. To verify the effectiveness of depth refined module, our experiment is conducted without the depth refined module. It is found that the ADD(S) increases by 15% under the refined depth maps. The depth refined module can reduce the depth error caused by discrete depth of the DORN. In the experiments, we find that the predicted depth can achieve 90.3% accuracy (within 5 cm) on objects in the YCB-video dataset.

Using the ground truth masks achieves better performance. Finally, we can also use the ground truth depth maps to obtain the initial *t*. This is also the upper limit of our method. The result is close to the state-of-the-art RGB-D methods [10].

### 4.4. Performance of Synthetic Depth Map

To evaluate the performance of synthetic depth maps introduced in Section 3.5, we show the results on the YCB-video dataset. With synthetic depth maps, the depth of objects becomes more accurate. Table 1 shows the result of synthetic depth maps. In contrast with results in Table 1, the performance of our model improves in every experiment by means of synthetic maps. This is because the original depth maps are not as accurate as the synthetic depth maps. Moreover, original depth maps lack depth in some areas. Therefore, This is an efficient method to generate a synthetic depth map for performance improvement.

### 4.5. Comparison with State-of-the-Art Methods

Our methods are compared with other state-of-the-art methods with RGB images as inputs and output 6D poses.

YCB-video dataset. PoseCNN is the first work that uses the YCB-video dataset. We use the same object segmentation module as PoseCNN, which ensures the fairness of the comparison in our experiment. We report the ADD AUC and the ADD-S AUC in Table 2. The AUC of our method is found to be much higher than that of PoseCNN [20] (ADD >15%, ADD-S >10%). Currently, DeepIM [30] uses the latest technology of YCB video. From Table 2, we find that our method performs better. In addition, we also compare our method with PVNet [28], which uses key points to figure out poses. PVNet does not present detailed scores in the YCB dataset. The PVNet obtains 73.4 for the average ADD(S) [28], which calculates the ADD AUC for asymmetric objects and the ADD-S AUC for symmetric objects. Our result is 78.5. As a result, we can state that our method achieves the current highest level on the YCB-video dataset.

Linemod dataset. The benchmarks of the Linemod dataset is different. Some research employs synthetic RGB images and refined methods [30,50] such as ICP. In this paper, we simply present the result without using refined methods. As shown in Table 3, it can be seen that our method is also effective in the Linemod dataset. CDPN[51] is the state-of-the-art method in the Linemod dataset. Our method has comparative performance with it.

T-LESS dataset. The metric in the T-LESS dataset is AR [52]. The result is shown in Table 4. We find that our network can achieve better performance than other methods. The T-LESS dataset has more symmetrical objects. The result proves that our network can deal with symmetrical objects well.

### 4.6. Symmetric Object Loss

In this paper, a new loss function for symmetric objects was put forward in Section 3.4. It can be observed from 2D projection images (Figure 5) that this new loss is able to handle particularly difficult cases. To evaluate the effectiveness of the new loss, we manifest the quantitative results of the comparison with the $l_{adds}$. From Figure 5, we find the ADD-S metric is not suitable to evaluate the performance of symmetric objects. The only way to fairly evaluate the performance of symmetric object loss is to rotate symmetric objects about their symmetric axes, and then obtain the actual performance with Formula (7). In this way, the nature of symmetry is considered, although the calculation becomes more difficult than the ADD-S. Let us call our new evaluation metric ADD-R for convenience.

**Figure 7:**
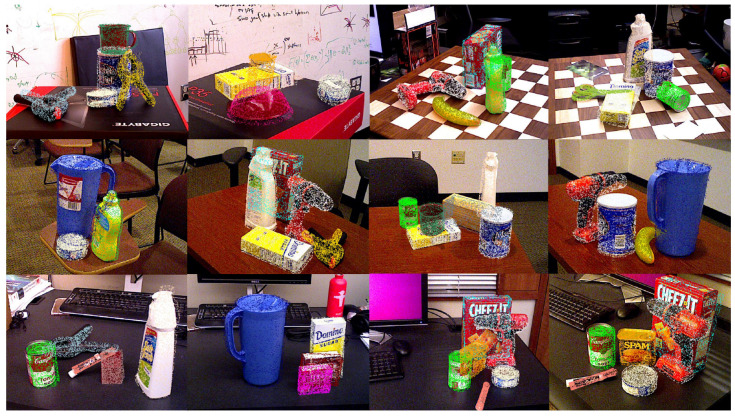
The visualization results of the 6D pose estimation.

Table 5 shows the quantitative results for symmetric objects of our model trained with $l_{addr}$ and $l_{adds}$. Due to the fact that the segmentation results of PoseCNN [20] cannot distinguish clamps and extra large clamps, we adopt ground truth masks in our experiments. Here we use the AUC the same method as in Section 4.3. The results of refined depth maps and ground truth depth maps are both given. Using $l_{addr}$, our result improves both in the ADD-S and the ADD-R. Our new loss achieves 3% improvement in the ADD-S. For the model trained with $l_{adds}$, bowls and clamps are particularly difficult cases. It is worth noting that although the frequency of bad cases is high in the test dataset, its ADD-S (AUC) is also high, which confirms the proposal that the ADD-S is not a suitable metric. The ADD-R (AUC) is smaller for these bad cases, and this is more reflective of the model’s performance on symmetric objects. The model trained with $l_{addr}$ significantly exceeds the model trained with $l_{adds}$ on the ADD-R (AUC). Figure 7 shows the visualization results of our pose estimation. This proves the effectiveness of our framework.

### 4.7. Time Efficiency

We present the run time of our framework to manifest its practicability. We evaluate our method on one 2080ti GPU. Reading data costs about 0.01 s for one frame. The segmentation module costs 0.03 s per frame. The translation estimation module costs 0.02 s to deal with an image, and the pose regression module costs 0.01 s per frame. In total, the run time of our method is up to 0.08 s per frame. More time comparisons are shown in Table 4. We refer to the time presented in the EPOS [55].

## 5. Conclusions

We put forward a 6D pose regression model with RGB images as inputs. A translation estimation module is designed to produce the accurate depth of objects, followed by the initial translations obtained through random sampling. There is a pose regression module calculating rotations and translation offsets to obtain the final poses. Moreover, we propose a new loss function for symmetric objects, which handles the particularly difficult cases caused by prior symmetric object loss. Experiments have verified the effectiveness of our approach, as described above.

## Figures and Tables

**Table 1:** Ablation Studies on the YCB-video Dataset. Nondepth is the result without using the translation estimation module. Nonrefine is the result without using the depth refined module. Refine is using the depth refined module. +gtmask uses ground truth masks. +gtdepth uses ground truth depth. GT is the result of using ground truth depth maps. SYN uses synthetic depth maps. Objects in bold are symmetric. We calculate the ADD Area under Curves (AUC) for non-symmetric objects and the ADD-S AUC for symmetric objects.

	Nondepth	NonRefine		Refine		+Gtmask		Gtmask+Gtdepth	
**Objects**	**Non**	**GT**	**SYN**	**GT**	**SYN**	**GT**	**SYN**	**GT**	**SYN**
002_master_chef_can	41.08	44.64	55.43	58.99	66.99	61.16	70.33	68.12	**71.79**
003_cracker_box	58.04	59.02	59.1	75.21	74.28	78.9	77.61	82.25	**83.52**
004_sugar_box	80.91	62.44	66.16	86.84	89	88.38	90.68	**94.55**	94.49
005_tomato_soup_can	65.45	59.77	60.56	79.84	83.17	80.61	85.3	87.4	**91.23**
006_mustard_bottle	80.07	67.73	67.44	87.52	86.5	88.8	87.66	**90.88**	89.0
007_tuna_fish_can	64.57	46.96	53.86	69.13	68.64	65.78	69.17	67.86	**70.96**
008_pudding_box	38.98	65.0	66.07	83.96	87.97	91.0	93.34	93.78	**93.8**
009_gelatin_box	58.67	64.45	64.16	86.29	89.36	90.86	94.44	**97.59**	96.58
010_potted_meat_can	62.14	48.45	59.6	72.97	79.16	74.67	80.98	76.19	**82.1**
011_banana	80.58	69.98	60.07	83.51	80.38	86.75	90.4	**92.0**	91.21
019_pitcher_base	83.25	57.56	84.1	86.78	77.96	88.13	77.97	**88.13**	87.56
021_bleach_cleanser	68.81	52.52	58.72	67.41	73.73	70.39	79.66	**89.93**	86.89
**024_bowl**	83.8	80.67	83.89	80.49	89.73	82.52	91.37	90.38	**93.82**
025_mug	56.76	44.63	62.22	71.94	85.5	63.68	86.88	76.43	**92.88**
035_power_drill	73.75	52.61	65.92	78.37	88.99	83.03	90.8	89.85	**92.58**
**036_wood_block**	69.54	63.54	73.03	66.43	77.5	70.4	82.71	86.38	**91.04**
037_scissors	39.06	43.7	38.2	63.14	55.39	67.37	47.91	**71.68**	56.92
040_large_marker	71.1	59.84	60.74	85.75	84.58	83.78	83.57	85.88	**85.99**
**051_large_clamp**	15.52	46.29	57.08	47.94	58.2	86.48	89.69	87.61	**92.69**
**052_extra_large_clamp**	21.4	46.88	55.65	54.43	59.86	71.57	80.36	71.08	**82.66**
**061_foam_brick**	74.87	87.44	86.9	87.88	91.38	87.64	92.2	92.4	**93.33**
AVG	61.35	58.29	63.76	74.99	78.5	79.14	83.0	84.78	**86.72**

**Table 2:** Quantitative Evaluation of 6D Pose on the YCB-Video Dataset. Objects in bold are symmetric.

	PoseCNN [20]		DeepIM [30]		SilhoNet [21]		Our	
**Objects**	**ADD**	**ADDS**	**ADD**	**ADDS**	**ADD**	**ADDS**	**ADD**	**ADDS**
002_master_chef_can	50.9	84	**71.2**	93.1	-	84	66.99	**93.35**
003_cracker_box	51.7	76.9	**83.6**	91	-	73.5	74.28	**92.53**
004_sugar_box	68.6	84.3	**94.1**	96.2	-	86.6	89	**96.85**
005_tomato_soup_can	66	80.9	**86.1**	92.4	-	88.7	83.17	**92.51**
006_mustard_bottle	79.9	90.2	**91.5**	95.1	-	89.8	86.5	**95.2**
007_tuna_fish_can	70.4	87.9	**87.7**	**96.1**	-	89.5	68.64	94.25
008_pudding_box	62.9	79	82.7	90.7	-	60.1	**87.97**	**93.89**
009_gelatin_box	75.2	87.1	71.9	**94.3**	-	92.7	**89.36**	93.81
010_potted_meat_can	59.6	78.5	76.2	86.4	-	78.8	**79.16**	**93**
011_banana	72.3	85.9	**81.2**	91.3	-	80.7	80.38	**92.37**
019_pitcher_base	52.5	76.8	**90.1**	**94.6**	-	91.7	77.96	91.37
021_bleach_cleanser	50.5	71.9	**81.2**	**90.3**	-	73.6	73.73	89.64
**024_bowl**	6.5	69.7	8.6	81.4	-	79.6	**30.97**	**89.73**
025_mug	57.7	78	81.4	91.3	-	86.8	**85.5**	**94**
035_power_drill	55.1	72.8	85.5	92.3	-	56.5	**88.99**	**94.04**
**036_wood_block**	31.8	65.8	60	**81.9**	-	66.2	**65.85**	77.5
037_scissors	35.8	56.2	**60.9**	75.4	-	49.1	55.39	**77.1**
040_large_marker	58	71.4	75.6	86.2	-	75	**84.58**	**91.85**
**051_large_clamp**	25	49.9	**48.4**	**74.3**	-	69.2	35.97	58.2
**052_extra_large_clamp**	15.8	47	31	**73.3**	-	72.3	**36.82**	59.86
**061_foam_brick**	40.4	87.8	35.9	81.9	-	77.9	**68.1**	**91.38**
AVG	53.7	75.9	71.7	88.1	-	79.6	**71.87**	**88.21**

**Table 3:** Quantitative Evaluation on the Linemod Dataset.

Methods	BB8 [53]	PoseCNN [20]	Pix2Pose [54]	PVNet [28]	CDPN [51]	Our
Mean	43.6	62.7	72.4	86.27	89.86	**90.34**

**Table 4:** Quantitative Evaluation on the T-LESS Dataset.

	EPOS [55]	CDPN [51]	Pix2Pose [54]	Sundermeyer [52]	Our
AR	47.6	12.4	27.5	30.4	**48.3**
Time(s)	0.75	0.67	0.81	0.19	**0.08**

**Table 5:** Contrast Experiments on Symmetric Object Loss.

	Refine+$l_{\mathrm{adds}}$		Refine+$l_{\mathrm{addr}}$		gtdepth+$l_{\mathrm{adds}}$		gtdepth+$l_{\mathrm{addr}}$	
**Objects**	**ADDS**	**ADDR**	**ADDS**	**ADDR**	**ADDS**	**ADDR**	**ADDS**	**ADDR**
024_bowl	85.16	45.12	91.37	83.2	86.78	48.09	**93.82**	**89.55**
036_wood_block	83.08	67.03	82.71	67.89	**91.17**	**83.2**	91.04	**83.2**
051_large_clamp	85.29	45.81	88.69	80.56	87.2	50.28	**92.69**	**88.18**
052_extra_large_clamp	76.43	50.13	80.36	84.13	78.17	53.06	**82.66**	**91.18**
061_foam_brick	93.34	43.75	92.2	72.25	**95.54**	44.26	93.33	**73.53**
AVG	84.66	50.37	87.07	77.61	87.77	55.78	**90.71**	**85.13**
